# Investigating the Challenges and Opportunities of Domiciliary Oral Care for the Older Adults: A Scoping Review

**DOI:** 10.3390/healthcare12232469

**Published:** 2024-12-06

**Authors:** Haslina Rani, Tuti Ningseh Mohd-Dom, Tew In Meei, Muhammad Syafiq Asyraf Rosli, Lee Zi Quan, Aznida Firzah Abdul Aziz, Siti Aisya Athirah Hassan, Nur Saadah Mohamad Aun

**Affiliations:** 1 Department of Family Oral Health, Faculty of Dentistry, Universiti Kebangsaan Malaysia, Kuala Lumpur 50300, Malaysia; hr@ukm.edu.my (H.R.); tutinin@ukm.edu.my (T.N.M.-D.); 2 Family Oral Wellness Research Group, Universiti Kebangsaan Malaysia, Kuala Lumpur 50300, Malaysia; inmeei@ukm.edu.my (T.I.M.); draznida@ppukm.ukm.edu.my (A.F.A.A.); 3 Department of Restorative Dentistry, Faculty of Dentistry, Universiti Kebangsaan Malaysia, Kuala Lumpur 50300, Malaysia; 4 Sungai Buloh Dental Clinic, Selangor State Health Department, Ministry of Health Malaysia, Sungai Buloh 47000, Malaysia; leeziquan1@gmail.com; 5 Department of Family Medicine, Faculty of Medicine, Universiti Kebangsaan Malaysia, Kuala Lumpur 56000, Malaysia; 6 Centre for Research in Psychology and Human Well-Being, Faculty of Social Sciences and Humanities, Universiti Kebangsaan Malaysia, Bandar Baru Bangi 43600, Malaysia; p144770@siswa.ukm.edu.my (S.A.A.H.); n_saadah@ukm.edu.my (N.S.M.A.)

**Keywords:** domiciliary care, elderly, oral health, caregiver

## Abstract

Background/Objectives: Older adults need specialised dental care due to age-related changes and chronic conditions, but inadequate coordination and capacity hinder access to domiciliary oral care. This review explores the characteristics, barriers, facilitators, caregiver education, and outcomes of domiciliary oral care to improve services for frail older adults. Methods: A systematic scoping search was conducted following the PRISMA guidelines. A literature search was performed to identify the key search terms and the databases that were relevant to the objectives. A total of 454 documents were retrieved, 31 of which were included in the final synthesis. Results: Overall, the barriers and facilitators in delivering domiciliary dental service for the elderly can be categorised into four groups: system, oral healthcare provider, caregiver, and patient. Having policies or guidelines supporting domiciliary oral care was one of the most frequently reported factors. Six studies reported outcomes of educational programme for caregivers and all were with positive results. Conclusions: The review highlights the need for a multi-pronged approach involving the healthcare system, oral healthcare providers, caregivers, and older adults themselves to improve access to and quality of oral healthcare services for this vulnerable population.

## 1. Introduction

As populations worldwide continue to age, the proportion of older adults is rapidly increasing. Globally, the number of people aged 60 and above is projected to double by 2050, accounting for more than 2 billion individuals [1]. This demographic shift presents significant challenges for healthcare systems, as older adults often face a higher burden of chronic diseases, physical frailty, and cognitive impairments. 

The decline, in general, of well-being and quality of life in older adults often leads to increased frailty, resulting in distinct dental care needs due to age-related changes, chronic conditions, mobility limitations, and cognitive impairments [2]. As populations age, the demand for domiciliary dental care—providing dental services outside traditional clinical settings, i.e., in a patient’s home or residential care facility—has grown significantly [3]. Domiciliary dental care is crucial for older adults who face physical, cognitive, or logistical barriers that prevent them from accessing dental clinics. Key services include preventive care, basic restorative procedures, denture-related treatments, palliative care, and oral health assessments. Delivery models encompass mobile dental clinics, portable dentistry, integrated care with healthcare providers, and community-based programmes. These services are offered in various settings, including private homes, nursing facilities, assisted living communities, and hospitals. This care model allows for greater flexibility, ensuring improved access to dental care for vulnerable populations and enhancing their quality of life.

However, despite the growing need for domiciliary dental care, many challenges may exist in delivering these services effectively. We hypothesise that a significant barrier is the lack of capacity among key stakeholders, including dentists, dental therapists, caregivers, healthcare personnel, and family members. The capacity gap can be attributed to inadequate training, education, and access to the appropriate dental equipment and materials, compounded by poor coordination and communication between stakeholders. This fragmentation of services hinders the ability to provide comprehensive oral care to older adults.

In addition, older adults who require domiciliary dental care often face numerous barriers, such as mobility issues, transportation challenges, and financial constraints, further limiting their access to essential dental services [4]. These obstacles will result in poor oral health outcomes, which can negatively affect their overall health, contributing to increased morbidity and reduced quality of life [5].

On a global scale, several initiatives have emerged to address these issues and improve domiciliary dental care services. Some countries have developed guidelines that include on-site treatments, caregiver training, and subsidised care, helping to reduce emergency visits and raise oral health awareness [6]. Integrating oral care into long-term care systems also reduces health issues, such as aspiration pneumonia, and enhances quality of life. However, challenges like resource shortages and inconsistent implementation limit the effectiveness of these strategies [6]. Furthermore, national health plans in various regions are placing greater emphasis on training oral health professionals to provide domiciliary care, recognising the importance of building a workforce capable of meeting the complex needs of the ageing population [7]. 

Significant gaps include a lack of clear policies, resource shortages, insufficient care-giver training, and challenges faced by patients, such as mobility limitations and financial constraints. These issues highlight a fragmented system unable to meet the comprehensive oral healthcare needs of the elderly. Hence, we hypothesise that addressing barriers to domiciliary oral care can significantly improve its access and quality, ultimately enhancing health outcomes for older adults.

By examining the challenges and opportunities for improvement, including enhanced training, inter-professional collaboration, and policy support, this scoping review aims to provide a global understanding of domiciliary oral care for older adults. Identifying these factors is crucial for developing strategies that improve access to quality dental care and address the growing oral health needs of ageing populations worldwide.

Review questions:

1. What are the characteristics and types of domiciliary oral care that have been implemented?
2. What are the barriers and facilitators in implementing domiciliary oral care?
3. What types of interventions are done for the caregivers, and what are the outcomes?

## 2. Materials and Methods

A scoping review was conducted following the PRISMA guidelines [8]. The Protocol for the scoping review was developed and registered with the OSF website (https://osf.io/s49tk/, accessed on 1 November 2023). In this scoping review, “older adults” or elderly is defined as individuals aged 60 years and above. This definition is in line with the definition given by the World Assembly on Ageing in 1982 in Vienna [9]. The included studies examined older adults aged 60 years and above (population) that lived in their own home or nursing home and received help from care providers, or home healthcare services (context). Studies that included younger age groups in addition to older adults were included if the data specific to those aged 60 years and above could be extracted. Furthermore, studies that addressed the implementation of different types of domiciliary oral care and outcomes for interventional studies (concept) were included. The search was limited to studies published in 2000 or later that are full-text papers. Research types like systematic or narrative reviews, protocol or tool development, as well as opinions or comments on the topic, were excluded.

The literature search was performed to identify the key search terms and the databases that were relevant to the objectives of the review on the following three electronic databases: PubMed, Scopus, and EBSCO. MeSH terms and keywords adapted to PubMed databases were used to ensure that all relevant literature is found with the search queries. The complete search strategy for the various databases is shown in Appendix A, Appendix B and Appendix C. 

Following the search, all identified citations were collated and uploaded into Rayyan.ai and duplicates were removed. Titles and abstracts were screened by three reviewers (LZQ, TNMD, HR) for assessment against the inclusion criteria for the review. Reasons for exclusion of sources that did not meet the inclusion criteria were recorded via labels in Google Sheets and reported in the scoping review. Potentially relevant sources were retrieved in full. The full text of selected citations was assessed in detail against the inclusion criteria by two reviewers independently (TNMD, HR). Disagreements that arise between the reviewers at each stage of the selection process were resolved through discussion with other research team members.

Data were extracted from papers included in the scoping review by three reviewers manually (LZQ, TNMD, HR). The data extracted included specific details about the key findings relevant to the research questions. Data extracted were analysed to seek emerging patterns.

## 3. Results

**Figure 1:**
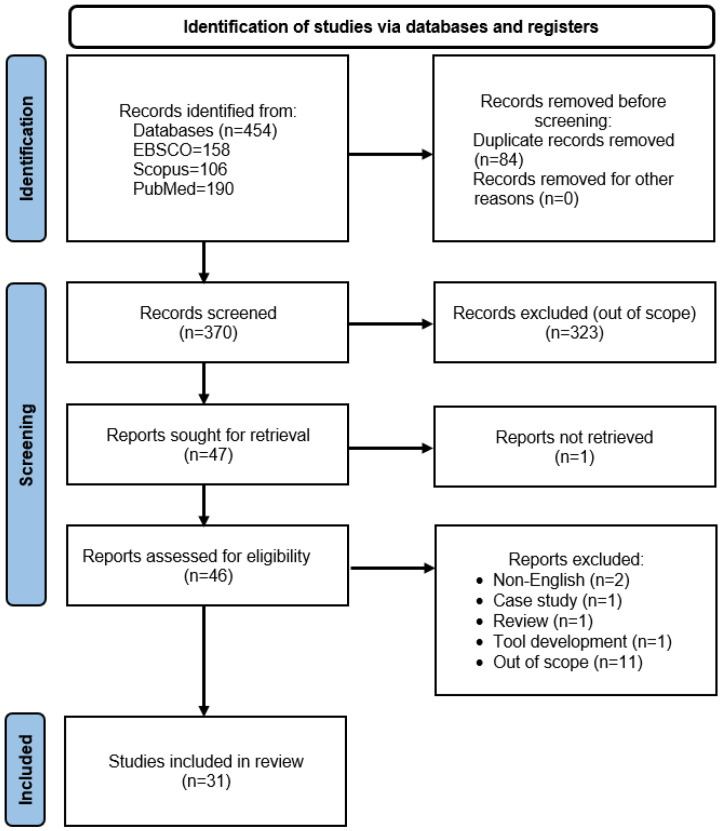
Selection of the literature following the PRISMA diagram (2020).

As displayed in Figure 1, a total of 31 studies were included in the final review following a rigorous protocol to retrieve studies that fit the scope of this review. Characteristics of each study are presented in Table 1. Only six studies reported interventions to look at the effectiveness of training caregivers on knowledge, attitude, and practice of oral health, while the rest of the studies were observational. Australia published the greatest number of studies (five), and six of the 31 studies analysed were conducted among dentists. Interventional studies did not report regular dental practice at the nursing homes where their studies were conducted. For the other studies, all studies reported some sort of oral care services provided for the elderly in their respective homes, mainly oral hygiene care and screening. Not many studies mentioned dental visits by dentists and fewer studies reported the details of dental treatment provided.

Analysing the facilitators in delivering domiciliary dental services for the elderly resulted in categorising them into four groups: system, oral healthcare provider, caregiver, and patient (Table 2). Facilitators related to the healthcare system were most frequently reported, with the highest number being on having guidelines or policies related to domiciliary care. This is followed by facilitating factors related to caregiver, such as training the caregivers on oral care.

Similarly, the same four categories were derived upon analysing the barriers reported (Table 3). Compared to the facilitators, there were more studies reporting barriers and challenges related to the provision of domiciliary dental care for the elderly. Most studies reported barriers related to healthcare systems and caregivers. Lack of facility or supplies and lack of clear policy or guidelines are the two most reported barriers related to the system. Meanwhile, for the caregiver category, lack of time dominated the list of barriers, followed by lack of knowledge and training related to oral care. A lot of studies also reported barriers that could be categorised into patient categories, with the most notable barriers being the lack of support from family members and poor attitude or behaviour related to oral health.

Table 4 summarises the interventions and outcomes of the interventions conducted. Only six studies were involved, and all interventions reported were educational programmes to train caregivers on how to care for the oral health of the elderly. All programmes involved educating the caregivers on the basics of oral care and knowledge on how to care for the elderly. Improved in knowledge was reported by three studies [10,11,12] and improved attitudes or behaviours were reported by three studies [10,11,12]. One study qualitatively reported the results where some caregivers were reluctant to use the new system introduced in the intervention programme, but some found it useful, relevant, and increased their awareness of the importance of oral care [13]. Only three studies looked at the oral health status parameters of the elderly in their respective studies. Three studies [12,14,15] reported improved plaque control of the elderly following education to the caregivers, and denture hygiene also showed significant improvement in two studies [12,14]. Denture stomatitis was significantly reduced after oral health education was provided to the caregivers [12], and improvement of mucosal plaque score was seen in another study among the independent elderly and the elderly with normal muscle strength [15].

**Table 1:** Characteristics of study and types of domiciliary care.

No (ID)	Author	Year	Study Design	Country	Category of Domicile	Elderly’s Heath Status	Type of Caregivers Studied	Types of Oral Care Mentioned
1	Smith M.B. et al. [16]	2017	Observational	New Zealand	Institutions and Own home	Not mentioned	Dentist	Dentists conducted visits to nursing homes. Oral care provided but type not specified
2	Hardgraves V.M. et al. [17]	2014	Observational	USA	Institutions	Vulnerable elders	Nursing staff	Oral care provided but type not specified
3	Tynan A. et al. [18]	2018	Observational	Australia	Institutions	Not cognitively impaired	Oral health therapist, nursing home staff	Screening, education, referral to dentist for remote real-time oral examination (teledentistry)
4	Catteau C. et al. [19]	2016	Observational	France	Institutions	Dependent elderly	Nurses and hospital agents	Oral hygiene care, two out of eight homes had visiting dentist, type of care not mentioned
5	Weening-Verbree L.F. et al. [20]	2021	Observational	Netherlands	Institutions	Frail	Nursing staff (nurses/ nurses aids) and managers	Oral hygiene care by nursing staff, 42% homes had visiting dentists, 8 of 12 homes had visiting dental hygienist. Type of oral care provided not specified.
6	Junges R. et al. [21]	2014	Observational	Brazil	Institutions	Independent elderly	Non-dental, non-familial caregivers but not specified	Oral hygiene care
7	Letchumanan D. et al. [22]	2020	Observational	Malaysia	Institutions	Varied, some bed-bound	Non-dental, non-familial caregivers but not specified	Dentists gave oral health education and tooth brushing technique, conducted examination and provided dental treatment at nursing home. Type of treatment not mentioned.
8	Zenthöfer A. et al. [14]	2016	Interventional/ controlled trial	Germany	Institutions	Severely dependent elderly, dementia	Non-dental, non-familial caregivers but not specified	Regular oral care practice not mentioned
9	Adebayo B. et al. [23]	2017	Observational	Australia	Institutions	Not mentioned	Non-dental, non-familial caregivers but not specified	Oral hygiene care
10	Janssens B. et al. [10]	2018	Interventional	Belgium	Institutions	Not mentioned	Nurses and nurse’s aids	Oral health assessment and an oral healthcare plan. Oral hygiene care based on the Oral healthcare Guideline for Older people in Long-term care institutions (OGOLI) and the daily oral healthcare protocol derived from the guideline. Preventive and curative dental care by visiting dentists.
11	Gerritsen P. et al. [24]	2015	Observational/ comparative	Netherlands	Institutions	Free from a severe illness	Dentists	Integrated dental care from check-up, hygiene care to dental treatment.
12	Hearn L. and Slack-Smith L. [25]	2016	Observational	Australia	Institutions	Not mentioned	Non-dental, non-familial caregivers but not specified	Oral hygiene care, limited visits by dentist type of care not mentioned
13	Ardenghi, D.M. and Wyatt, C. [26]	2017	Observational	Canada	Institutions and Own home	Dependent elderly	Familial caregivers	Screening and simple dental care
14	Solchiro H. et al. [27]	2013	Observational	Japan	Institutions and Own home	Not mentioned	Dentists	Around 30% dentists provided domiciliary dental care, type of care not mentioned
15	Jones R.J. et al. [28]	2018	Observation	Wales	Institutions	Good mental capacity, some had physical limitations	Care home manager and non-healthcare givers	Oral hygiene care provided at home. Residents need to seek dental treatment at dental facilities outside.
16	Kerr E. et al. [29]	2022	Observational	Northern Ireland	Institutions and Own home	Dependent, Bed-ridden	Dentists	Domiciliary dental care provided by dentists, types of care not mentioned
17	Gomez-Rossi J. et al. [30]	2022	Observational	Germany	Institutions	Varied, some bed-bound	Care home managers, section managers, nurses and dentists	Oral hygiene care. Domiciliary dental care by dentists but type of care not mentioned.
18	Konstantopoulou K. et al. [11]	2021	Interventional	Greece	Institutions	Frail and care dependent	Non-dental, non-familial caregivers but not specified	Regular oral care practice not mentioned
19	Khanagar S. et al. [12]	2015	Interventional/ cluster randomised trial	India	Institutions	Totally dependent	Non-dental, non-health, non-familial caregivers	Regular oral care practice not mentioned
20	Othman A.A. et al. [31]	2014	Observational	Malaysia	Institutions and Own home	Frail and dependent elderly	Government dentists	36% experienced providing domiciliary dental care Main treatment provided- check-up, health education, simple extraction and denture related procedures.
21	Cornejo-Ovalle M. et al. [32]	2013	Observational	Spain	Institutions	Not mentioned	Nursing assistants	Oral hygiene care
22	Edman K. and Wardh I. [33]	2022	Observational	Sweden	Institutions	Not mentioned	Licenced nurse, assistant nurse, managers	Oral hygiene care
23	Stephenson, M. H. G. et al. [34]	2018	Observational	New Zealand	Institutions	Not mentioned	Nurses, non-healthcare givers, managers	Oral hygiene care Indirect dental referrals with either GP referrals or family contact
24	Portella F. et al. [15]	2015	Interventional	Brazil	Institutions	Good cognitive status	Non-dental, non-familial caregivers but not specified	Regular oral care practice not mentioned
25	Wagner S. et al. [13]	2022	Mixed-method	Denmark	Institutions	Not mentioned	Non-dental, non-familial caregivers but not specified	Regular oral care practice not mentioned
26	Palmers E. et al. [35]	2022	Observational	Belgium	Institutions and Own home	Not mentioned	Nursing home managers and caregivers (nurse, nurse aid, speech therapist, social worker, physiotherapist, general practitioner)	Oral hygiene care
27	Ohara Y. et al. [36]	2021	Observational	Japan	Own home	Home-bound	Familial caregivers	Oral hygiene care
28	Delgado A.M. et al. [37]	2016	Observational	USA	Institutions	Not mentioned	Non-dental, non-familial caregivers but not specified	Oral hygiene care
29	Webb B.C. et al. [38]	2015	Observational	Australia	Institutions	Not mentioned	Directors of nursing at aged care facilities	Oral hygiene care
30	Webb B.C. et al. [39]	2013	Observational	Australia	Institutions	Functionally dependent	Directors of nursing at aged care facilities	Emergency dental treatment—73.4% Dentists visit nursing home regularly—25.8% Oral hygiene care
31	Kamijo S. et al. [40]	2018	Observational	Japan	Institutions and Own home	Not mentioned	Dentists who practised domiciliary dental care	Removable prosthetic—96% Oral hygiene care—91% Dysphagia rehabilitation—65% Periodontal treatment- 62% Restorative treatment—54% Surgical treatment—51%

**Table 2:** Facilitators for domiciliary care.

Category	Facilitator	Study ID
System	Having guidelines or policy on domiciliary oral care for elderly	[13,20,22,31,32,35,40]
	Collaboration between care facilities, dental and other healthcare providers	[27,30]
	Having in-house oral care provider	[20]
Oral healthcare provider	Having positive attitude towards domiciliary care for elderly	[29]
	Received training on domiciliary oral care for elderly	[27]
Caregiver	Received training on oral care for elderly	[20,32]
	Having good knowledge on oral care for elderly	[30]
	Having experience in oral care for elderly	[33]
Patient	Positive attitude towards oral health	[20,40]
	Having good family support	[28]

**Table 3:** Barriers to domiciliary care.

Category	Barriers	Study ID
System	Lack of facility or supplies	[10,16,17,20,30,31,39]
	Lack of or unclear policy or guidelines focusing on domiciliary care for elderly	[16,18,23,29,30,35]
	Lack of manpower (caregiver)	[16,19,23,35]
	Poor/lack of communication/coordination or collaboration between nursing care facilities and oral healthcare facilities/provider	[17,18,23,25]
	Less ergonomic working condition	[31,35]
	Financial/cost related issues	[18,25]
	High demand/high workload	[31]
	Lack of appropriate dental equipment	[18]
Oral healthcare provider	Lack of time/not priority	[17,23]
	Lack of experience/not prepared	[16,18]
	Reluctance in treating elderly	[25]
	High risk of litigation	[29]
	Lack of financial incentives	[29]
	Lack of training on how to provide domiciliary dental care	[29]
	Lack of knowledge on how to provide domiciliary dental care	[17]
	Lack of skills in providing domiciliary dental care	[18]
Caregiver	Lack of time/not priority	[14,16,17,18,19,20,21,23,34,35,36]
	Lack of knowledge on how to provide domiciliary dental care	[10,11,16,20,22,30,34,35,36]
	Lack of training on how to provide domiciliary dental care	[11,19,23,25,29,31,34]
	Lack of financial incentives/low salary/financial concerns	[10,17,23,25,39]
	Lack of skills in providing domiciliary dental care	[11,16,30,35]
	Unfavourable attitude towards oral care	[10,16,20,31]
	Lack of experience	[18,30]
Patient	Lack of family support	[10,18,25,31,34,39]
	Poor attitude or behaviour related to oral health	[19,22,23,24,31,39]
	Financial concerns	[10,13,16,17,34]
	Lack the ability to perform self-care	[15,16,19,24,25]
	Uncooperative to care	[21,23,30]
	Communication issue	[13,22,38]
	Complex medical condition	[29,31]
	Lack of knowledge	[16,22]

**Table 4:** Interventions conducted.

No (ID)	Intervention	Knowledge Outcome	Behavioural/Attitude Outcome	Health Status
8 [14]	1. Two-day comprehensive education programme for caregivers of participants with and without dementia. The contents of the programme include: - PowerPoint presentation on age-related changes, pathologies of the oral cavity, standardised estimation tools of oral conditions, feasible toothbrushing techniques, handling of interdental space brushes and mouth rinses for caregivers and use of ultrasound baths for denture cleaning. - Video on oral care - Training on use of revised oral assessment guide - Training on handling of different kinds of removable dentures 2. Use of ultrasonic baths to clean dentures. 3. Module on Communication with physicians	n.a	n.a	Plaque control record (PCR) at follow-up for intervention group improved from 85.7% to 70.1% (*p* < 0.001). No significant improvement for control group. Denture hygiene index (DHI) at follow-up for intervention group improved from 84.5% to 57.8% (*p* < 0.001). No significant improvement for control group.
10 [10]	Implementation of daily oral healthcare protocol derived from the OGOLI guideline to the administrators of the nursing homes, caregivers and oral healthcare practitioners. Guideline consists of theory, practical and dental visit to deliver preventive and curative oral care to the residents.	Knowledge for the intervention group improved significantly higher compared to the improvement of knowledge among the control group which was (*p* < 0.001)	Attitude for the intervention group improved significantly (*p* < 0.001)	n.a
18 [11]	Geriatric oral health education programme- theory and practical (90 min session). The content of the programme was based on the recommendations by the European College of Gerodontology and the European Geriatric Medicine Society on the expected knowledge and skills of non-dental healthcare professionals about oral health promotion in frail older adults.	Total knowledge score for intervention group is significantly higher than total knowledge score for control group after delivery of oral health education programme (*p* < 0.001)	Total attitude score for intervention group is significantly higher than total knowledge score for control group after delivery of oral health education programme (*p* = 0.017)	n.a
19 [12]	Oral-health education was provided to the caregivers of the study group via PowerPoint presentation at baseline. CD and manual of the oral care were also provided. Reinforcement was provided to the study group at 3-month follow-up. Oral-health education to the caregivers of control group was given at the end of six months. Content of oral health education programme was not mentioned.	Significant improvement in the oral-hygiene knowledge of the caretakers of the study group, whereas there was no improvement among the caretakers of the control group. There was significant improvement in knowledge on importance of oral health and oral hygiene, use of fluorides, denture care and denture hygiene practices, management of dry mouth, and importance of regular dental check-ups.	Significantly improved oral-hygiene practices of the elderly residents in the study group after educating their caretakers	Debris index in study group improved from 2.87 ± 0.22 to 1.49 ± 0.34 (*p* < 0.001). Plaque index in study group improved from 3.17 ± 0.40 to 1.57 ± 0.35 (*p* < 0.001). Denture plaque in study group improved from 3.15 ± 0.47 to 1.21 ± 0.27 (*p* < 0.001). Denture stomatitis in study group improved from 1.43 ± 0.68 to 0.29 ± 0.52 (*p* < 0.001). No significant difference was seen in control group for all variables reported.
24 [15]	The intervention consisted of an educational programme and specific guidelines for caregivers (to perform oral hygiene for dependent elderly and to supervise the independent elderly during oral hygiene practices)	n.a	n.a	Improved plaque score and mucosal score of elderly
25 [13]	Introduction of a basic oral care adherence aid system (as a reminder to caregivers to brush the teeth of elderly and a mean to register brushing efforts)	n.a	Some caregivers were reluctant to use a new system but some caregivers find the system is useful, relevant and increased their awareness on the importance of oral care.	n.a

n.a—not applicable.

## 4. Discussion

This scoping review comprehensively analysed the challenges and opportunities in providing domiciliary oral care for older adults, emphasising systemic-, caregiver-, provider-, and patient-related factors. The findings highlight the multifaceted nature of domiciliary care, underscoring the need for an integrated and patient-centred approach to overcome barriers and improve access.

*Characteristics of domiciliary oral care*

Table 1 provides an overview of the characteristics and types of domiciliary care described in the included studies, highlighting the variability in care provision. While many studies focused on basic oral hygiene and education, few addressed comprehensive dental treatments like restorative care or denture management [41]. Comprehensive care is essential for not just for detecting and managing dental problems early, but also to help restore oral functions of the elderly involved and prevent further complications. Further, only a limited number of studies reported regular dentist visits to nursing homes or domiciliary settings, with most interventions centred on caregiver-led oral hygiene. The variability in study contexts—ranging from private homes to institutional settings—also emphasises the importance of tailoring interventions to the specific needs of different care environments.

*Systemic Barriers and Facilitators*

The lack of clear policies and guidelines emerged as a critical systemic barrier to effective domiciliary oral care. Without standardised frameworks, implementation is inconsistent and fragmented, leading to disparities in service delivery. Conversely, the presence of supportive policies and collaborative networks among healthcare providers may facilitate better integration of oral care into broader healthcare systems. For instance, integrating oral health into national health plans and providing subsidies or insurance coverage have proven effective in some settings [41]. Future strategies should focus on developing cohesive policies that recognise domiciliary oral care as an essential component of geriatric healthcare.

*Caregiver Training and Support*

Caregivers play a central role in domiciliary oral care, especially for older adults with cognitive or physical impairments. Educational interventions in this study reported improvements in caregiver knowledge, attitudes, and practices [10,11,12,13,14,15]. These interventions led to better plaque control, denture hygiene, and overall oral health outcomes for older adults. However, the sustainability of these outcomes is limited by caregiver challenges, such as time constraints, lack of financial incentives, and insufficient support. Addressing these challenges through structured caregiver education programmes, financial incentives, and ongoing support can enhance their capacity to provide consistent and effective care.

*Provider Challenges*

Oral healthcare providers too face significant barriers in the delivery of domiciliary oral care for the elderly. This includes the lack of experience in domiciliary settings, time constraints, and inadequate equipment. Training programmes tailored to domiciliary care can help build provider capacity and confidence. Additionally, inter-professional collaboration models may effectively address barriers to oral care for the elderly in community settings by integrating diverse healthcare professionals to deliver patient-centred care that meets complex geriatric needs. This approach is especially important in geriatric care, where oral health is often overlooked. An interdisciplinary team approach, involving dentists, physicians, nurses, and social workers, enables tailored geriatric assessments and care plans [42]. Programmes like “Don’t forget the mouth!” in the Netherlands have demonstrated the effectiveness of interprofessional care in improving oral health among community-dwelling frail older people [43]. An important team member in community setting which is often overlooked in dental care is a social worker. Social workers are trained to provide care coordination, emotional support, and caregiver assistance, an essential role in domiciliary healthcare [41]. This role can be further expanded to include advocacy and coordination of domiciliary dental care for home-bound elderly.

*Patient-Related Factors*

Patients’ attitudes, beliefs, and behaviours were also identified as important factors influencing the provision of domiciliary oral care. Financial constraints, poor attitudes toward oral health, and lack of family support emerged as critical barriers to seeking dental care for older adults. Many older adults deprioritise oral health due to misconceptions about its importance later in life or a focus on immediate comfort over long-term care [43]. Recognising the significant barriers to oral healthcare—such as physical limitations, cognitive impairments, and financial constraints—it is crucial that services also address the individual needs, preferences, and challenges of older adults. Addressing these challenges requires patient-centred strategies that consider socio-economic and cultural contexts. Hence, financial support, such as subsidies or insurance coverage, and educational initiatives tailored to individual preferences can motivate older adults and their families to prioritise oral health.

*Future Directions*

The findings highlight the need for a multi-pronged approach to improve domiciliary oral care. This includes the development of robust policies, enhanced caregiver and provider training, and innovative care models that integrate oral health into general healthcare systems. Future research should evaluate the long-term impact of these interventions and explore scalable solutions to meet the growing oral health needs of ageing populations globally. By addressing barriers across all levels—systemic, caregiver, provider, and patient—this review highlights actionable strategies to enhance domiciliary oral care. Implementing these strategies can significantly improve access and quality of care, ultimately enhancing the health and well-being of older adults.

## 5. Conclusions

This scoping review provides a comprehensive overview of the current evidence on the challenges and opportunities in delivering domiciliary oral care for community-dwelling older adults. The review highlights the need for a multi-pronged approach involving the healthcare system, oral healthcare providers, caregivers, and older adults themselves to improve access to and quality of oral healthcare services for this vulnerable population. 

## Data Availability

The protocol for this study is publicly available at https://osf.io/s49tk/ (accessed on 1 November 2023). The results are presented in the result section and Appendix A, Appendix B and Appendix C of this paper.

## Appendix A

**Table A1:** PUBMED Search terms.

Search Terms (Pubmed)			Total Results
	#1	“Home Care Services”[MeSH Terms] OR “Residential Facilities”[MeSH Terms] OR “Long-Term Care”[MeSH Terms]	127,554
	#2	“domiciliary care”[Title/Abstract] OR “domiciliary health care”[Title/Abstract] OR “home care”[Title/Abstract] OR “home health care”[Title/Abstract] OR “care dependent”[Title/Abstract] OR “nursing home”[Title/Abstract] OR “home nursing”[Title/Abstract] OR “residential facility”[Title/Abstract] OR “residential care”[Title/Abstract] OR “institutionalized”[Title/Abstract] OR “in-house”[Title/Abstract] OR “long-term care”[Title/Abstract] OR “homes for the aged”[Title/Abstract] OR “home for the aged”[Title/Abstract]	111,925
	#3	#1 OR #2	190,410
	#4	Aged[MeSH Terms]	3,468,011
	#5	“Older”[Title/Abstract] OR “elder*”[Title/Abstract] OR “aged”[Title/Abstract]	1,382,982
	#6	#4 OR #5	4,255,545
	#7	“Dental Prophylaxis”[MeSH Terms] OR “Dental Health Services”[MeSH Terms:noexp] OR “Dental Care”[MeSH Terms:noexp] OR “Dental Care for Aged”[MeSH Terms] OR “Oral Health”[MeSH Terms] OR “Oral Hygiene”[MeSH Terms]	67,805
	#8	“dental prophylaxis”[Title/Abstract] OR “oral prophylaxis”[Title/Abstract] OR “dental care”[Title/Abstract] OR “oral care”[Title/Abstract] OR “dental health”[Title/Abstract] OR “oral health”[Title/Abstract] OR “dental cleaning”[Title/Abstract] OR “oral cleaning”[Title/Abstract] OR “dental hygiene”[Title/Abstract] OR “oral hygiene”[Title/Abstract] OR “professional brushing”[Title/Abstract] OR “professional teeth cleaning”[Title/Abstract] OR “professional cleaning”[Title/Abstract]	68,969
	#9	#7 OR #8	104,319
	#10	”Dental Auxiliaries”[MeSH Terms] OR ”Dentists”[MeSH Terms] OR ”Dental Staff”[MeSH Terms]	34,380
	#11	“dental auxiliar*”[Title/Abstract] OR “dentist*”[Title/Abstract] OR “dental nurse*”[Title/Abstract] OR “dental assistant*”[Title/Abstract] OR “dental hygienist*”[Title/Abstract] OR “dental professional*”[Title/Abstract] OR “professional oral care”[Title/Abstract] OR “professional oral health”[Title/Abstract] OR “professional dental care”[Title/Abstract] OR “professional dental health”[Title/Abstract] OR “domiciliary oral health”[Title/Abstract] OR “domiciliary dental care”[Title/Abstract] OR “dental staff”[Title/Abstract] OR “dental personnel”[Title/Abstract]	97,084
	#12	“caregiver*” OR “carer*” OR “care provider*” OR “health aide” OR “helper*” OR “guardian*”	274,981
	#13	#10 OR #11 OR #12	388,570
	#14	#3 AND #6 AND #9 AND #13	755
	#15	#3 AND #6 AND #9 AND #13 <filters: full-text, 2000 to present>	190

## Appendix B

**Table A2:** Scopus search terms.

Search Terms (Scopus)			Total Results
	#1	“domiciliary care” OR “domiciliary health care” OR “home care” OR “home health care” OR “care dependent” OR “nursing home” OR “home nursing” OR “residential facility” OR “residential care” OR “institutionalized” OR “in-house” OR “long-term care” OR “homes for the aged” OR “home for the aged”	434,081
	#2	“older” OR “elder*” OR “aged”	6,785,391
	#3	dental prophylaxis” OR “oral prophylaxis” OR “dental care” OR “oral care” OR “dental health” OR “oral health” OR “dental cleaning” OR “oral cleaning” OR “dental hygiene” OR “oral hygiene” OR “professional brushing”	146,713
	#4	“dental auxiliar*” OR “dentist*” OR “dental nurse*” OR “dental assistant*” OR “dental hygienist*” OR “dental professional*” OR “professional oral care” OR “professional oral health” OR “professional dental care” OR “professional dental health” OR “domiciliary oral health” OR “domiciliary dental care” OR “dental staff” OR “dental personnel” OR “dental therapist”	202,535
	#5	“caregiver*” OR “carer*” OR “care provider*” OR “health aide” OR “helper*” OR “guardian*”	378,215
	#6	#1 AND #2 AND #3 AND #4 AND #5	117
	#7	#1 AND #2 AND #3 AND #4 AND #5 <filter: 2000 to present>	106

## Appendix C

**Table A3:** EBSCO search terms.

Search Terms (EBSCO-Dentistry and Oral Sciences)			Total Results
	#1	“domiciliary care” OR “domiciliary health care” OR “home care” OR “home health care” OR “care dependent” OR “nursing home” OR “home nursing” OR “residential facility” OR “residential care” OR “institutionalized” OR “in-house” OR “long-term care” OR “homes for the aged” OR “home for the aged”	2287
	#2	“older” OR “elder*” OR “aged” OR “geriatric”	21,955
	#3	“dental prophylaxis” OR “oral prophylaxis” OR “dental care” OR “oral care” OR “dental health” OR “oral health” OR “dental cleaning” OR “oral cleaning” OR “dental hygiene” OR “oral hygiene” OR “professional brushing” OR “professional teeth cleaning” OR “professional cleaning”	42,452
	#4	“dental auxiliar*” OR “dentist*” OR “dental nurse*” OR “dental assistant*” OR “dental hygienist*” OR “dental professional*” OR “professional oral care” OR “professional oral health” OR “professional dental care” OR “professional dental health” OR “domiciliary oral health” OR “domiciliary dental care” OR “dental staff” OR “dental personnel” OR “dental therapist*”	67,597
	#5	“caregiver*” OR “carer*” OR “care provider*” OR “health aide” OR “helper*” OR “guardian*”	3589
	#6	#4 OR #5	70,055
	#7	#1 AND #2 AND #3 AND #5	158
